# The prophylactic antiemetic therapies in management of differentiated thyroid cancer patients with radioactive iodine therapy: a single-center, non-randomized clinical trial

**DOI:** 10.3389/fendo.2024.1310223

**Published:** 2024-04-19

**Authors:** Xiao Li, Jingjia Cao, Wenxiu Wang, Xiaolu Zhu, Yaru Sun, Lei Song, Wei Zhang, Yong Han

**Affiliations:** ^1^ Department of Nuclear Medicine, the Second Hospital of Shandong University, Jinan, China; ^2^ School of Basic Medical Sciences, Shandong University, Jinan, China; ^3^ Department of Radiology, the Second Hospital of Shandong University, Jinan, China; ^4^ Department of Thyroid Surgery, Binzhou Medical University Hospital, Binzhou, China

**Keywords:** differentiated thyroid cancer, nausea, vomiting, antiemetic, ondansetron

## Abstract

**Objective:**

The present study was to investigate three different single-drug regimens to show which was more effective to reduce radioactive iodine therapy (RAI) associated nausea and vomiting, and to compare the occurrence of long-term gastrointestinal diseases after RAI therapy.

**Method:**

We performed a single-center, non-randomized clinical trial among patients who underwent RAI therapy from March 2016 to July 2022. Enrolled patients were divided into four cohorts based on the date of the treatment. cohort 1, with no preventive antiemetics; cohort 2, received 20 mg of pantoprazole per day for 3 days; cohort 3, received a 10 mg metoclopramide tablet two times daily for 3 days; cohort 4, oral ondansetron, 8 mg, twice daily for 3 days. The primary endpoints were proportion of patients who experience vomiting episodes and nausea during the 7-day hospital period. Secondary end points included Functional Living Index Emesis (FLIE) quality-of life questionnaires and the occurrence of gastrointestinal diseases.

**Results:**

A total of 1755 patients were analyzed, comprised of 1299 (74.0%) women and 456 (26.0%) men, with a median age of 44 years (range 18–78 years). The characteristics of patient were similar within the four groups. 465 (26.4%) patients developed RAI-associated nausea, and 186 (14.4%) patients developed RAI-associated vomiting. The rate of nausea was significantly decreased in the patients who were taking ondansetron when compared with the other cohorts (*P*<0.05), while the rate of vomiting (≥6 episodes) was slightly lower. As secondary endpoint, FLIE measures ondansetron scored highly compared to other cohorts, from baseline (mean score of 110.53 ± 17.54) to day 7 (mean score of 105.56 ± 12.48). In addition, 48 (2.7%) patients were found to be with gastrointestinal diseases at the end of one year follow up. Multiple RAI therapy and higher dose of I-131 per body weight revealed a significantly independent risk factors of developing gastrointestinal disorders.

**Conclusions:**

In conclusion, the present study demonstrated that short-term ondansetron could be an effective prophylactic agent in controlling RAI-associated nausea and vomiting. Furthermore, the risk of developing gastrointestinal disorders was significantly higher for patients with multiple RAI therapy and higher dose of I-131 per body weight.

## Introduction

In recent years, the incidence and prevalence of differentiated thyroid carcinoma (DTC) has steadily risen in worldwide (1). As an important postoperative adjuvant therapy, radioactive iodine (RAI) therapy was performed to remove residual thyroid tissue and potential residual thyroid cancer cells to decrease the rate of disease recurrence (2). Although RAI therapy was tolerated well by most patients, taking a certain dose of radioactive iodine-131 orally may cause gastrointestinal (GI) toxicities in clinical practice (3). RAI-associated nausea and vomiting is one of the commonest immediate side effects during hospitalization when no prophylactic antiemetic is given, affecting up to 65% of the patients, and may have adverse effects on their quality of life (QoL) (4). Therefore, recognizing the RAI-associated nausea and vomiting and using antiemetic agents appropriately could improve patients’ subjective experience, thereby leading to better QoL.

It is postulated that, similar to the mechanism of external beam radiation, RAI would stimulate the gastrointestinal mucosa to release 5-hydroxytryptamine 3 (5-HT3), leading to nausea and vomiting (5). Additionally, the literature showed that gastrointestinal (GI) toxicities associated with radiation exposure were caused by oxidative stress, and it could cause GI damage through numerous pathways (6). According to the oncology antiemetic guidelines, it is recommended to use 5-HT 3 receptor antagonists prophylactically for patients receiving low to high risk external radiation therapy for vomiting (7). Obviously, compared to a complete course of external radiation therapy, the radiation dose of RAI therapy to the GI is usually lower. There is little information on the measures to RAI-associated nausea and vomiting, and the prophylaxis may be ignored.

The conventional antiemetics, such as dopamine receptor antagonists (e.g., metoclopramide), proton pump inhibitors (e.g., pantoprazole) are commonly used for the management of RAI therapy to alleviate gastrointestinal toxicities. A report proposed by Mirza et al. has shown that the ondansetron was considered superior to conventional antiemetic agents (8). However, there are few published records in large randomized trials comparing 5-HT3 receptor antagonists with conventional antiemetics or placebo for the treatment of radiation induced symptoms. Furthermore, gastrointestinal exposure to radioactive iodine-131 causes long-term comorbidity or not remained unclear to our knowledge. a recent study by Lee et al. found that RAI therapy was related to an increased risk of gastrointestinal disorders, when the cumulative dose exceeds 1.11 GBq (4). The present study was to investigate three different single-drug regimens to show which was more effective to reduce radioactive iodine therapy (RAI) associated nausea and vomiting, and to compare the occurrence of long-term gastrointestinal diseases after RAI therapy.

## Materials and methods

### Study design

**Figure 1:**
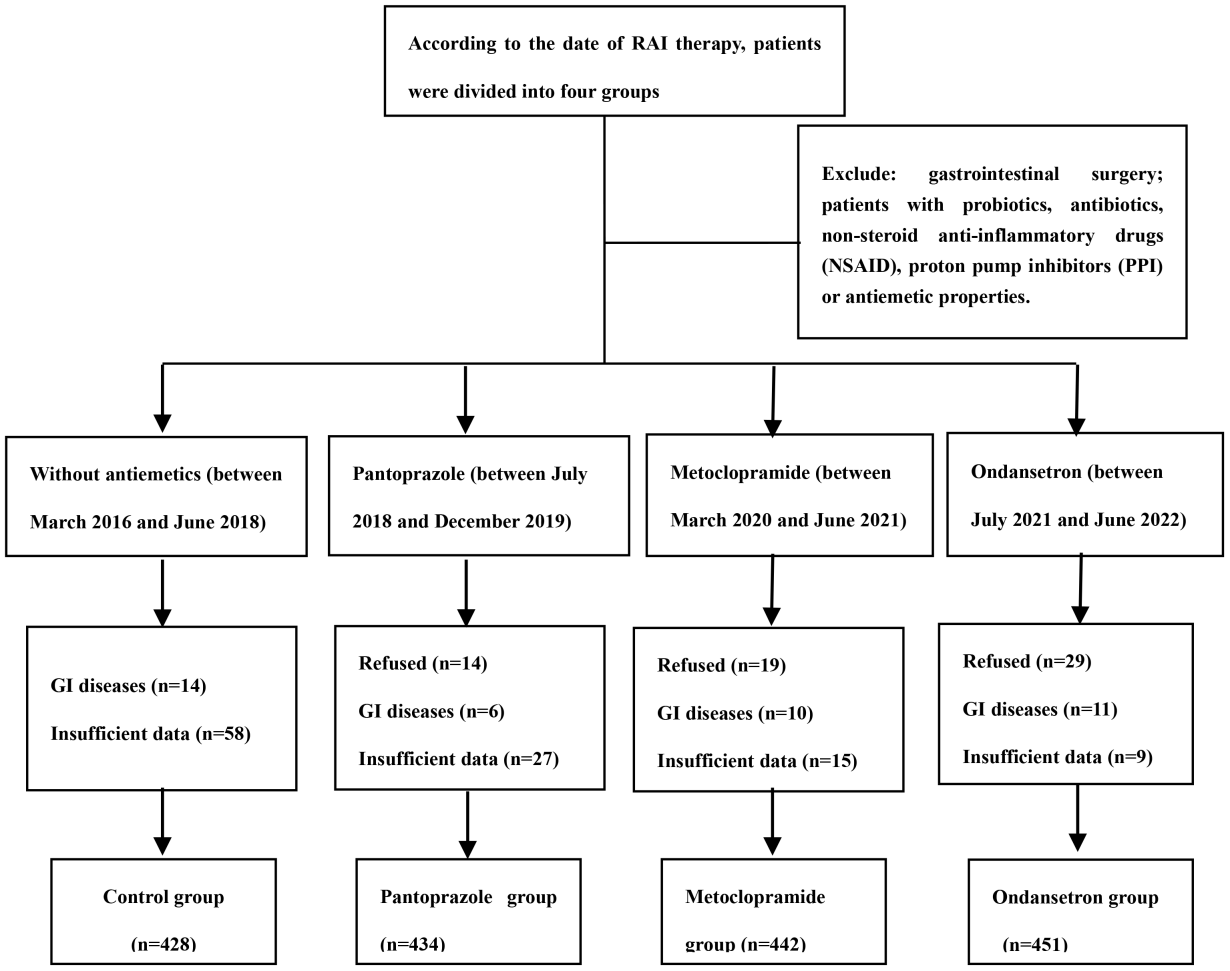
The enrollment flow chart.

We extracted the data from the medical records from March 2016 to July 2022 to identify the study population. Eligible patients were adults (aged 18 to 80 years) who were able to provide consent forms and were received RAI treatment after thyroid surgery. Patients with obvious gastrointestinal diseases, patients with a history of gastrointestinal surgery and patients with long-term use of any probiotics or antibiotics, non-steroid anti-inflammatory drugs (NSAID) or proton pump inhibitors (PPI) were excluded from the study. We assumed the incidence of RAI-associated nausea to be approximately 50% after RAI therapy. Thus, the sample of 400-500 patients in each group provided 90% power at the 95% level of significance. The flowchart is shown in **Figure 1**. Ethical approval was obtained from the Ethics Committee of the Second Hospital of Shandong University (Jinan, China).

### Intervention protocol

Enrolled patients were divided into four cohorts based on the date of the treatment. The groups characteristics were as follows: Cohort 1, control group, between March 2016 and June 2018, routine radioiodine therapy with no preventive antiemetics; Cohort 2, intervention, between July 2018 and December 2019, received 20 mg of pantoprazole per day for 3 days, beginning on the day of I-131 therapy; Cohort 3, intervention, between March 2020 and June 2021, received a 10 mg metoclopramide tablet two times daily before RAI therapy for 3 days; Cohort 4, intervention, between July 2021 and June 2022, oral ondansetron, 8 mg, was administered 1–2 h before RAI therapy, twice daily for 3 days thereafter as maintenance treatment. In order to reduce the incidence of bias and confounding factors, all antiemetics used are from the same brand of each medication.

### The RAI therapy protocol

A month before RAI, patients were asked to withdrawn L-Thyroxine 4 (LT4) treatment and follow a low-iodine diet. Routine medical examinations included thyroid-stimulating hormone (TSH), pre-ablation stimulated thyroglobulin (ps-Tg), TgAb and neck ultrasound (US) were performed just before initial RAI. TSH >30 “μIU/ml “ was required after thyroid hormone withdrawal (THW). According to 2015 ATA risk stratification, patients had ablation orally with doses of RAI ranged from 1.11 GBq (30 mCi) to 5.55 GBq (150 mCi).

Evaluate responses through diary cards and questionnaires filled out by patients and research nurses. The daily diary evaluated their daily vomiting and nausea, as well as their use of antiemetics. The possible impact of clinical-pathological characteristics (age, gender, TNM stage of tumor) and response to RAI therapy was acquired from medical records. Based on the World Health Organization (WHO) classification, patients were divided into four groups: underweight (BMI<18.5 kg/m^2^), normal weight (18.5-24.9 kg/m^2^), overweight (25-29.9 kg/m^2^), and obese (BMI≥30 kg/m^2^). Dose per BW was expressed as the dose of RAI administered (MBq) divided by body weight (kg).

### Evaluation of subjective symptoms

The primary endpoints were proportion of patients who did not experience vomiting episodes and nausea during the 7-day hospital period. According to the National Cancer Institute’s Common Terminology Criteria for Adverse Events (CTCAE) version 4.0, nausea grade comprised of mild, moderate and severe. A single episode of vomiting was defined as vomiting and/or retching separated by intervals of 5 min or less. Antiemetic clinical trials often use complete response, partial control and uncontrolled response as an endpoint. The definition of complete response refers to no emetic episodes or use of rescue medication. Partial control was defined as 1–5 episodes of vomiting, but no use of rescue medication. Uncontrolled response was defined as ≥6 episodes of vomiting.

In addition, the impact of RAI-associated nausea and vomiting on patients’ quality of life was evaluated using the Functional Life Index-Emesis (FLIE). The FLIE questionnaire includes two parts: nausea (Q1–9) and vomiting (Q10–18) (9). Patients completed the FLIE at baseline, days 3 and 7 during treatment. If the total score is>108 points, it is considered to have little impact on the quality of life; On the contrary, if the total score is ≤ 108 points, it is considered that RAI-associated nausea and vomiting has a negative impact on the patient’s life. FLIE was considered as secondary observation indicator.

### The occurrence of gastrointestinal disorders

At the end of follow up, the diagnosis of gastrointestinal diseases, such as gastric ulcers, duodenal ulcers, and atrophic gastritis, was also documented as a secondary observation indicator.

### Statistical analysis

The results are expressed as mean ± SD for continuous data and percentage (%) for categorical data. Four group were compared using the χ2-test or Fisher’s exact test for categorical variables or the Kruskal-Wallis test for continuous variables. Univariate and multivariate logistic regression analyses were performed to evaluate the association between the development of RAI-associated nausea and vomiting, gastrointestinal diseases and the clinical parameters. All the statistical analyses were performed using SPSS 22.0 software, with statistical significance defined as *P*<0.05.

## Results

### Baseline characteristics

A total of 1755 patients were analyzed after excluding 245 patients for the following reasons: 62 patients did not receive the allocated intervention in time, 41 patients had gastrointestinal diseases, and 142 patients without sufficient follow-up data. The patients comprised of 1299 women and 456 men, with a median age of 44 years (range 18–78 years). Of the total cohort, 7.1% (125/1755) patients underwent lobectomy, and the other patients underwent near or total thyroidectomy. 228 (12.9%) in the low and 994 (56.6%) in the intermediate risk of recurrence patients, respectively. Patients received a median dose of 3700 MBq (range1110–7400 MBq). The history of smoking and alcohol consumption were found in 197 (11.2%) and 253 (14.4%) of patients, respectively. One hundred and sixty-five patients were diagnosed with moderate and severe anxiety by self-rating anxiety scale.

In cohort 1, between March 2016 and June 2018, 428 patients were analyzed. In cohort 2, between July 2018 and December 2019, 434 patients were analyzed. In cohort 3, between March 2020 and June 2021, 442 patients were analyzed. In cohort 4, between July 2021 and June 2022, 451 patients were analyzed. The difference in distribution of these characteristics was not statistically significant from the control group for all of the intervention groups. Specifically, there were no significant differences in the distribution of age, gender, BMI, administered dose, ATA risk of recurrence, history of smoking, alcohol consumption, and the number of RAI treatments among the four included cohorts (**Table 1**).

**Table 1:** Characteristics of patients who underwent RAI therapy with different anti-emetics.

	Control group (n=428)	Pantoprazole group (n=434)	Metoclopramide group (n=442)	Ondansetron group (n=451)	*P*
**Age (years)**	44.0 (34.0, 54.0)	44 (34.0, 53.0)	45.0 (34.0, 55.0)	44.0 (34.0, 54.0)	0.40
<55	326 (76.2%)	329 (75.8%)	324 (73.3%)	341 (75.6%)	0.75
≥55	102 (23.8%)	105 (24.2%)	118 (26.7%)	110 (24.4%)	
Gender (n, %)					
Male	121 (28.3%)	109 (25.1%)	104 (23.5%)	122 (27.1%)	0.39
Female	307 (71.7%)	325 (74.9%)	338 (76.5%)	329 (72.9%)	
BMI (n, %)					
<18.5 kg/m^2^	77 (18.0%)	70 (16.1%)	78 (17.6%)	72 (16.0%)	0.98
18.5-24.9 kg/m^2^	131 (30.6%)	144 (33.2%)	148 (33.5%)	151 (33.5%)	
25-29.9 kg/m^2^	152 (35.5%)	154 (35.5%)	155 (35.1%)	159 (35.2%)	
≥30 kg/m^2^	68 (15.9%)	66 (15.2%)	61 (13.8%)	69 (15.3%)	
**History of smoking (n, %)**	45 (10.5%)	56 (12.9%)	49 (11.1%)	47 (10.4%)	0.63
**Alcohol consumption (n, %)**	67 (15.7%)	64 (14.7%)	54 (12.2%)	68 (15.1%)	0.48
Type of surgery (n, %)					
Lobectomy	30 (7.0%)	22 (5.1%)	41 (9.3%)	32 (7.1%)	0.12
Total thyroidectomy	398 (93.0%)	412 (94.9%)	401 (90.7%)	419 (92.9%)	
SAS at baseline (n, %)					
Normal and mild anxiety	388 (90.7%)	384 (88.5%)	404 (91.4%)	414 (91.8%)	0.37
Moderate and severe anxiety	40 (9.3%)	50 (11.5%)	38 (8.6%)	37 (8.2%)	
ATA stratification (n, %)					
Low	59 (13.8%)	56 (12.9%)	59 (13.3%)	54 (12.0%)	0.04
Intermediate	256 (59.8%)	252 (58.1%)	222 (50.2%)	264 (58.5%)	
High	113 (26.4%)	126 (29.0%)	161 (36.5%)	133 (29.5%)	
**Cumulative ^131^I activity (MBq)**	3700 (1850, 5550)	3700 (2960, 5550)	3700 (1850, 7400)	3700 (1850, 7400)	0.92
RAI therapy (n, %)					
Single	335 (78.3%)	384 (88.5%)	372 (84.2%)	377 (83.6%)	0.01
Multiple	93 (21.7%)	50 (11.5%)	70 (15.8%)	74 (16.4%)	
**Dose per BW (MBq/kg)**	47.6 ± 15.6	47.6 ± 17.6	48.3 ± 13.5	47.8 ± 14.6	0.56
Biochemical Results					
TSH (lIU/mL)	112.0± 49.0	111.3± 48.1	111.7± 46.5	111.9± 47.8	0.98
Thyroglobulin levels (ng/mL)	2.6 (1.2,7.1)	1.6 (0.6,3.8)	1.1 (0.4,1.9)	1.7 (0.6,4.1)	0.01

SAS, self-rating anxiety scale; BMI, body mass index; BW, body weight; TSH, thyroid stimulating hormone; Dose per BW, the dose of RAI administered (MBq) divided by body weight (kg).

### Efficacy endpoints

**Figure 2:**
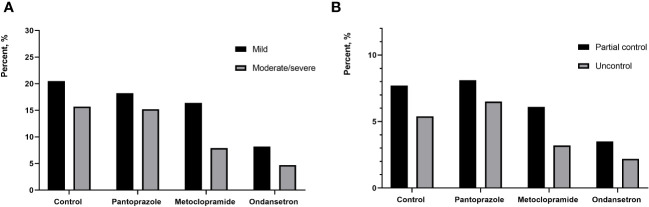
**(A)** percentage of patients reporting mild and moderate/severe nausea between different groups; **(B)** percentage of patients reporting partial control and uncontrol between different groups.

**Figure 3:**
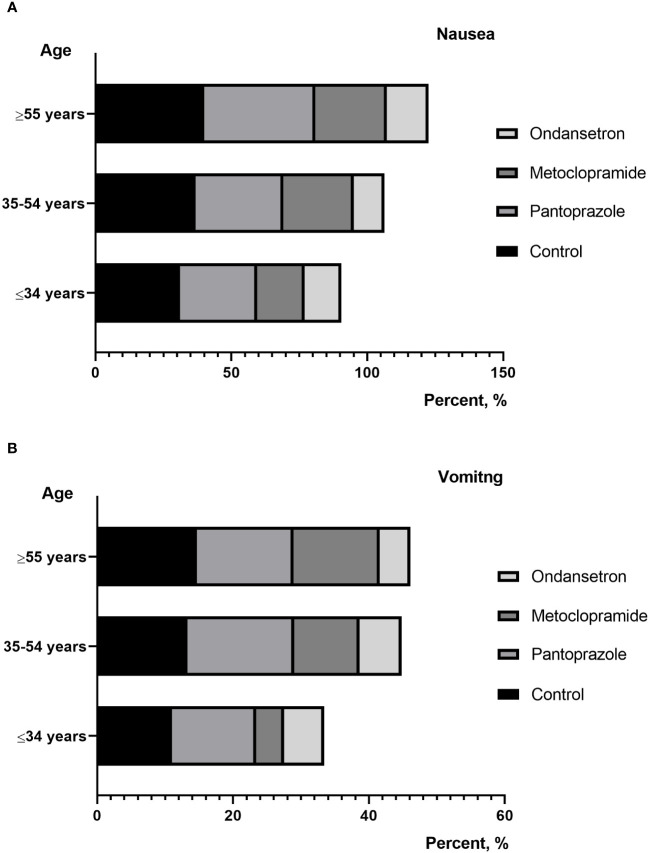
**(A)** percentage of patients reporting nausea between different age; **(B)** percentage of patients reporting vomiting between different age.

In total, 465 (26.4%) patients who developed nausea associated with RAI therapy. Of these, 276 (15.7%) patients had mild nausea, 189 (10.7%) patients had moderate and severe nausea. Additionally, RAI-associated vomiting was observed in 186 (14.4%) patients. The nausea was recorded in 36.3%, 33.3%, 24.2% and 12.9% of cohort 1 to 4 patients, respectively. Compared to other cohort, the lowest incidence of vomiting was in the cohort that used ondansetron. Moderate/severe nausea was also significantly less frequent in the ondansetron group with 4.7% reporting, while 15.2% of the pantoprazole group reported moderate/severe nausea. The vomiting was recorded in 13.1%, 14.6%, 9.3% and 5.7% of cohort 1 to 4 patients, respectively. Ondansetron was found to be with a rate of 94.3% for complete control. In contrast, only 90.3% of patients treated with metoclopramide experienced the same level of control (*P*=0.01) (**Figures 2**, **3**). The other drug, pantoprazole, did not control vomiting.

As a secondary endpoint, the relationship between nausea and vomiting-related items in FLIE at follow-up was studied. Although there was a decrease in nausea and vomiting scores from the baseline (mean score of 110.53 ± 18.26) to day 7 (mean score of 99.33 ± 24.70) for patients in the control group, there was little change in the quality-of-life assessments for patients in the ondansetron group from baseline (mean score of 110.53 ± 17.54) to day 7 (mean score of 105.56 ± 12.48). Although these are subjective, measures ondansetron scored highly compared to other cohorts.

At the end of one year follow up, 48 (2.7%) patients were found to be with gastrointestinal diseases, which comprised of 10 patients with gastric ulcers, 4 patients with duodenal ulcers, and 34 patients with atrophic gastritis, respectively. Treatment responses are summarized in **Table 2**.

**Table 2:** The primary and second endpoints of clinical outcomes between different groups.

	Control group (n=428)	Pantoprazole group (n=434)	Metoclopramide group (n=442)	Ondansetron group (n=451)	*P* value
Nausea, n (%)					
Mild	88 (20.5%)	79 (18.2%)	72 (16.4%)	37 (8.2%)	0.01*
Moderate/severe	67 (15.7%)	61 (15.2%)	35 (7.9%)	21 (4.7%)	
Acute Nausea, n (%)					
Mild	22 (5.1%)	16 (3.7%)	18 (4.1%)	9 (2.0%)	0.01*
Moderate/severe	19 (4.4%)	21 (4.8%)	10(2.3%)	7 (1.6%)	
Delayed Nausea, n (%)					
Mild	66 (15.4%)	63 (14.5%)	54 (12.3%)	28 (6.2%)	0.01*
Moderate/severe	48 (11.3%)	40 (10.4%)	25 (5.6%)	14 (3.1%)	
Vomiting, n (%)					
Partial control	33 (7.7%)	35 (8.1%)	27 (6.1%)	16 (3.5%)	0.01*
Uncontrol	23 (5.4%)	28 (6.5%)	14 (3.2%)	10 (2.2%)	
FLIE score					
At baseline	110.53 ± 18.26	109.13 ± 17.16	109.47 ± 18.26	110.53 ± 17.54	0.56
Day 3	100.13 ± 20.14	100.35 ± 19.44	102.13 ± 19.47	106.16 ± 13.18	0.01*
Day 7	99.33 ± 24.70	99.87 ± 24.70	101.83 ± 22.46	105.56 ± 12.48	0.01*
Gastrointestinal diseases, n (%)					
Atrophic gastritis	12 (2.8%)	9 (2.1%)	8 (1.8%)	5 (1.1%)	NA
Gastric ulcers	3 (0.7%)	2 (0.4%)	4 (0.9%)	1 (0.2%)	NA
Duodenal ulcers	2 (0.4%)	1 (0.2%)	1 (0.2%)	0	NA

FLIE, Functional Living Index Emesis; acute nausea (within 3 days) and delayed nausea (days 3–7); NA, indicates not available.

The bold label means statistically significant difference; *, means statistical significance (Control vs Ondansetron, Pantoprazole vs Ondansetron, Metoclopramide vs Ondansetron).

### Adverse events

3 and 27 patients reported headache and constipation, respectively. All incidences of headache were mild. No significant adverse drug reactions were reported from the four included groups.

### Risk factors of RAI-associated nausea and vomiting

In univariate and multivariate logistic regression analysis, the development of nausea and vomiting was significantly associated with moderate/severe anxiety (*P*=0.01), lower BMI (*P*=0.01), and multiple treatments (*P*=0.01). Furthermore, we also found that patients who developed nausea and vomiting had significantly higher dose of I-131 per body weight (*P*=0.01). Meanwhile, among the evaluated variables, younger age and total thyroidectomy were associated with nausea in univariate logistic regression analysis. However, in multivariate logistic regression analysis, there is no statistical difference between them (**Table 3**).

**Table 3:** Univariate and multivariate logistic regression analysis of clinical parameters between patients who developed nausea and vomiting in RAI therapy.

	Univariate						Multivariate					
	Nausea			Vomiting			Nausea			Vomiting		
	*OR*	(95% CI)	*P*	*OR*	(95% CI)	*P*	*OR*	(95% CI)	*P*	*OR*	(95% CI)	*P*
**Age (years)**	1.01	1.00-1.01	**0.01**	1.01	0.99-1.01	0.26	1.01	0.98-1.02	0.52	1.03	0.97-1.02	0.81
<55	Ref.			Ref.			Ref.			Ref.		
≥55	1.29	1.01 = 1.64	**0.03**	1.13	0.80-1.59	0.48	0.99	0.61-1.61	0.99	1.01	0.47-2.16	0.96
**Sex (female)**	0.82	0.64-1.04	0.10	0.86	0.61-1.21	0.41	1.37	1.01-1.85	**0.03**	0.81	0.51-1.29	0.38
**History of smoking**	0.93	0.66-1.31	0.71	0.88	0.54-1.46	0.64						
**Alcohol consumption**	0.91	0.68-1.26	0.64	0.96	0.62-1.48	0.85						
Type of surgery												
Lobectomy	Ref.			Ref.			Ref.			Ref.		
Total thyroidectomy	0.23	0.16-0.33	**0.01**	0.43	0.27-0.70	**0.01**	0.71	0.29-1.29	0.21	0.83	0.43-1.62	0.59
SAS at baseline												
Normal/mild	Ref.			Ref.			Ref.		Ref			
Moderate/severe	27.1	16.8-43.5	**0.01**	64.5	42.1-98.6	**0.01**	31.3	18.5-53.1	**0.01**	60.6	38.5-95.4	**0.01**
BMI												
<18.5 kg/m^2^	Ref.			Ref.			Ref.			Ref.		
18.5-24.9 kg/m^2^	1.04	0.68-1.61	0.83	0.70	0.40-1.23	0.22	1.11	0.78-1.58	0.54	1.31	0.73-2.37	0.36
25-29.9 kg/m^2^	4.33	2.91-6.42	**0.01**	0.39	0.23-0.66	**0.01**	0.21	0.13-0.31	**0.01**	0.34	0.17-0.69	**0.03**
≥30 kg/m^2^	0.24	0.15-0.37	**0.01**	1.52	1.00-2.30	**0.04**	0.20	0.11-0.33	**0.01**	0.76	0.35-1.62	**0.01**
ATA stratification												
Low	Ref.			Ref.								
Intermediate	1.25	0.89-1.74	0.19	1.32	0.81-2.16	0.25						
High	1.07	0.74-1.54	0.71	0.95	0.55-1.63	0.86						
RAI therapy												
Single	Ref.			Ref.			Ref.					
Multiple	5.26	4.03-6.87	**0.01**	3.25	2.33-4.54	**0.01**	5.46	3.94-7.55	**0.01**	2.34	1.45-3.7	**0.01**
**Dose per BW**	3.24	2.33-4.35	**0.01**	2.46	1.39-3.89	**0.01**	4.38	1.34-5.45	**0.01**	1.54	0.76-3.11	**0.01**
Biochemical Results												
TSH levels	1.01	0.99-1.01	0.81	1.01	0.99-1.01	0.55						
Thyroglobulin levels	0.92	0.57-1.45	0.53	0.91	0.62-1.56	0.79						

BMI, body mass index; SAS, self-rating anxiety scale; ATA, American Thyroid Association; Dose per BW, the dose of RAI administered (MBq) divided by body weight (kg).

The bold label means statistically significant difference.

### Risk factors of gastrointestinal diseases

In univariate logistic regression analysis, the cumulative risk of developing gastrointestinal disorders was significantly higher for patients with multiple RAI therapy, higher dose of I-131 per body weight and received ondansetron. After adjustment for multivariate logistic regression analysis, multiple RAI therapy and higher dose of I-131 per body weight revealed a significantly independent risk factors of developing gastrointestinal disorders (*OR*=17.3, *P*=0.01; *OR*=1.62, *P*=0.03) (**Table 4**).

**Table 4:** Risk factors analysis for gastrointestinal disorders using logistic regression.

	Univariate			Multivariate		
	*OR*	(95% CI)	*P*	*OR*	(95% CI)	*P*
**Age**	1.01	0.98-1.02	0.56			
**Sex(female)**	0.57	0.31-1.04	0.06	0.73	0.33-1.65	0.45
**History of smoking**	1.86	0.88-3.91	0.09	1.31	0.47-3.68	0.61
**Alcohol consumption**	1.38	0.686-2.89	0.38			
Type of surgery						
Lobectomy	Ref.					
Total thyroidectomy	0.52	0.21-1.25	0.14			
SAS at baseline						
Normal/mild	Ref.					
Moderate/severe	1.67	0.73-3.79	0.21			
BMI						
<18.5 kg/m^2^	Ref.					
18.5-24.9 kg/m^2^	1.66	0.71-3.91	0.24			
25-29.9 kg/m^2^	0.73	0.27-1.95	0.53			
≥30 kg/m^2^	1.12	0.39-3.26	0.82			
ATA stratification						
Low	Ref.					
Intermediate	0.73	0.33-1.65	0.46			
High	0.74	0.31-1.79	0.51			
RAI therapy						
Single	Ref.			Ref.		
Multiple	17.4	8.93-33.9	**0.01**	17.3	8.85-34.1	**0.01**
**Dose per BW**	2.83	1.32-3.46	**0.02**	1.62	1.32-2.89	**0.03**
Biochemical Results						
TSH levels	1.01	0.99-1.01	0.97			
Thyroglobulin levels	0.97	0.76-1.32	0.85			
Anti-emetics						
Control	Ref.			Ref.		
Pantoprazole	0.68	0.32-1.45	0.32	1.04	0.46-2.37	0.91
Metoclopramide	0.73	0.35-1.52	0.41	0.94	0.43-2.05	0.88
Ondansetron	0.32	0.12-0.83	**0.04**	0.38	0.14-1.01	0.05

BMI, body mass index; SAS, self-rating anxiety scale; ATA, American Thyroid Association; Dose per BW, the dose of RAI administered (MBq) divided by body weight (kg).

The bold label means statistically significant difference.

## Discussion

In 1953, radiation-induced nausea and vomiting was firstly described by Brown (10). The early gastrointestinal toxicity after external beam radiation therapy has been confirmed in the literature. According to the dosimetry studies proposed by Brill et al., the highest radiation dose is absorbed by the stomach, bladder, and salivary glands in the external thyroid organs. The retention, secretion and absorption of radioactive iodine in gastric mucosa is the sources of gastrointestinal radiation exposure after RAI therapy (11). Therefore, we have researched the efficacy of the prophylactic administration in management of DTC undergoing RAI therapy.

Nausea is the most common gastrointestinal symptom in RAI therapy, but not often accompanied by vomiting. We established a control group who did not receive prophylactic antiemetics. In our study, RAI-associated nausea was recorded in 36.3% of patients in this group. A prospective study of DTC patients who received 5.55 GBq of I-131 showed that 50% complained of nausea, which was higher comparable to our study. This may be due to the fact that the initial dose of most of our patients is significantly lower than 5.55 GBq. However, the incidence of nausea has been reduced to varying degrees since the use of prophylactic drugs in the present study. The incidence of RAI related nausea in patients taking pantoprazole and metoclopramide was 33.4% and 24.2%, respectively. Similarly, Kita et al. reported that the incidence of RAI-associated nausea in patients receiving domperidone treatment was 40.2%, which is also higher than our study (12). Obviously, proton pump inhibitors (e.g., pantoprazole) used for the management of RAI therapy to alleviate nausea had a mild effect. researchers assumed that the pantoprazole effectively reduce gastric acid pro- duction and increase gastric pH, would decrease the tissue inflammation resulting from radioiodine ingestion but the current study did not demonstrate this to be useful. In this study, ondansetron has also been shown to be more effective in controlling vomiting than other traditional antiemetics. Vomiting was known to be more difficult to control than nausea. When considering vomiting in patients without any antiemetic, RAI-associated vomiting was documented in 13.1%. 94.3% of patients in the ondansetron group had a complete response to therapy (no emesis and no rescue medication), while the proportion of patients in the without antiemetic group was 86.9% (*P*=0.01). Compared with metoclopramide, the frequency of vomiting was lower after prophylaxis with ondansetron (5.7% *vs* 9.3%).

The likelihood that a patient receiving radiation therapy will experience nausea and vomiting has been difficult to quantify. In the absence of quality of life (QoL) tool for RAI-associated nausea and vomiting, Functional Living Index-Emesis (FLIE) was used, which was originally aimed at measuring the impact of chemotherapy on cancer patients. In the present study, the average FLIE score was 110.9 ± 23.5 at baseline, indicating that there was no or minimal impact on daily life after thyroid surgery. There were differences in the overall QoL among four cohorts at Day 3 and Day 7. Patients in the ondansetron group had better scores in the domains of nausea and vomiting when compared with patients in others group. Similarly, Sykes et al. obtained conclusions when used the FLIE QoL questionnaires to compare the different group before therapy and at the end of study. It is postulated that patients in the ondansetron group reported better functioning, the overall QoL and lower symptom levels.

Individual risk factors for nausea and vomiting caused by external radiation include age, gender, alcohol consumption, anxiety, and previous experiences of nausea and vomiting (13, 14). In the present study, we found that patients with lower BMI, moderate/severe anxiety were more susceptible to nausea and vomiting in RAI therapy. Further research is needed to elucidate the reasons for the anxiety-dependent development of RAI-associated nausea. However, age, gender, history of smoking or drink and ATA risk status did not show any correlation. Conversely, Kita et al. reported that RAI-associated gastrointestinal diseases was significantly associated with younger female. It has been speculated that young female patients may have an enhanced physiologic response to a variety of emetic stimulants (15). Interestingly, we also found that the higher RAI dose per body weight was associated with RAI-associated nausea and vomiting. In addition, as the number of treatments increases, the dosage of I-131 taken by the patient increases, and the incidence of gastrointestinal reactions and vomiting gradually increases. This may be due to the increase in the dosage of I-131 treatment, which increases the intensity of radioactive stimulation in the patient’s stomach. Nausea may be a non-specific complaint and may have causes other than RAI therapy such as hypothyroidism. In patients with multiple RAI therapy prepared with THW, prolonged hypothyroidism was induced. The nausea and vomiting increased meaningfully in the patients with a TSH elevation (12).Therefore, laboratory parameters including TSH levels after thyroid hormone withdrawal were recorded. However, the present study shown that TSH levels was not associated with nausea and vomiting.

Compared to a complete course of external radiation therapy, the radiation dose of RAI therapy to the stomach is usually lower. To our knowledge, there is currently insufficient evidence of gastrointestinal diseases caused by radiation exposure to RAI therapy (16, 17). Of course, not all patients completed a long-term flowed-up about gastrointestinal disorders, making interpretation difficult. However, from the available data it appeared to be related to the treatment frequency (*P*=0.01) and RAI dose per body weight (*P*=0.03). Lee et al. reported that the average time to develop a gastrointestinal disorder was 3.82 ± 3.01 years after RAI therapy (4). However, the gastrointestinal diseases mentioned in his study already was included constipation, which was reasonable and inconsistent with us. The gastric absorption proposed by Johansson et al., dose per unit of RAI activity is 1.2 mGy/MBq (18). In clinical practice, the empiric RAI therapy dose may lead to gastric doses of approximately 1.3-8.9 Gy. It is postulated that RAI induces nausea and vomiting might be sufficient to cause long-term mucosal damage to the stomach and duodenum. Among the four cohorts, the patients with ondansetron had lower incidence rate of gastrointestinal diseases in our follow-up. However, potentially inappropriate medications (PIMs) are highly prevalent in the elderly and may have adverse effects on health-related outcomes (19).

This study has several limitations. First, the patients in the four cohorts were not randomized, and unknown confounding factors may interfere with the results of the study. Second, considering the mild symptoms of nausea and vomiting, a single drug was chosen in this study. Actually, the combination use of antiemetics can be more effective in preventing RAI related nausea. Third, although the large sample size may compensate for this deficiency, the single center nature of the study may limit our analysis. Hence, for correctly evaluating the effect of prophylactic antiemetics on the treatment of RAI, a longer follow-up study may be needed.

In conclusion, the present study demonstrated that short-term ondansetron could be an effective prophylactic agent in controlling RAI-associated nausea and vomiting. Furthermore, the risk of developing gastrointestinal disorders was significantly higher for patients with multiple RAI therapy and higher dose of I-131 per body weight.

## Data availability statement

The raw data supporting the conclusions of this article will be made available by the authors, without undue reservation.

## Ethics statement

The studies involving humans were approved by the Ethics Committee of the Second Hospital of Shandong University (KYLL-2022(LW)078). The studies were conducted in accordance with the local legislation and institutional requirements. Written informed consent for participation in this study was provided by the participants’ legal guardians/next of kin.

## Author contributions

JC: Writing – original draft. XL: Writing – original draft. WW: Visualization, Writing – review & editing. XZ: Writing – review & editing. YS: Writing – review & editing. WZ: Writing – review & editing. LS: Writing – review & editing. YH: Writing – review & editing.
